# A *Villin*-Driven *Fxr* Transgene Modulates Enterohepatic Bile Acid Homeostasis and Response to an *n*-6-Enriched High-Fat Diet

**DOI:** 10.3390/ijms21217829

**Published:** 2020-10-22

**Authors:** Spencer N. Wren, Micah G. Donovan, Ornella I. Selmin, Tom C. Doetschman, Donato F. Romagnolo

**Affiliations:** 1 Department of Nutritional Sciences, The University of Arizona, Tucson, AZ 85721, USA; swren@uab.edu (S.N.W.); selmin@arizona.edu (O.I.S.); 2 Interdisciplinary Cancer Biology Graduate Program, The University of Arizona, Tucson, AZ 85724, USA; mdono123@email.arizona.edu; 3 The University of Arizona Cancer Center, Tucson, AZ 85724, USA; 4 Department of Cellular and Molecular Medicine, The University of Arizona, Tucson, AZ 85724, USA; tdoetsch@arizona.edu

**Keywords:** farnesoid X receptor, bile acids, high-fat diet, soybean oil, *n*-6, linoleic acid

## Abstract

A diet high in *n*-6 polyunsaturated fatty acids (PUFAs) may contribute to inflammation and tissue damage associated with obesity and pathologies of the colon and liver. One contributing factor may be dysregulation by *n*-6 fatty acids of enterohepatic bile acid (BA) metabolism. The farnesoid X receptor (FXR) is a nuclear receptor that regulates BA homeostasis in the liver and intestine. This study aims to compare the effects on FXR regulation and BA metabolism of a palm oil-based diet providing 28% energy (28%E) from fat and low *n*-6 linoleic acid (LA, 2.5%E) (CNTL) with those of a soybean oil-based diet providing 50%E from fat and high (28%E) in LA (*n*-6HFD). Wild-type (WT) littermates and a transgenic mouse line overexpressing the *Fxrα1* isoform under the control of the intestine-specific *Villin* promoter (*Fxrα1^TG^*) were fed the CNTL or *n*-6HFD starting at weaning through 16 weeks of age. Compared to the CNTL diet, the *n*-6HFD supports higher weight gain in both WT and *Fxrα^TG^* littermates; increases the expression of *Fxr*α*1/2*, and peroxisome proliferator-activated receptor-γ*1* (*Pparγ1*) in the small intestine, *Fxrα1/2* in the colon, and cytochrome P4507A1 (*Cyp7a1*) and small heterodimer protein (*Shp*) in the liver; and augments the levels of total BA in the liver, and primary chenodeoxycholic (CDCA), cholic (CA), and β-muricholic (βMCA) acid in the cecum. Intestinal overexpression of the *Fxra1^TG^* augments expression of *Shp* and ileal bile acid-binding protein (*Ibabp)* in the small intestine and *Ibabp* in the proximal colon. Conversely, it antagonizes *n*-6HFD-dependent accumulation of intestinal and hepatic CDCA and CA; hepatic levels of *Cyp7a1*; and expression of *Pparγ* in the small intestine. We conclude that intestinal *Fxrα1* overexpression represses hepatic de novo BA synthesis and protects against *n*-6HFD-induced accumulation of human-specific primary bile acids in the cecum.

## 1. Introduction

The consumption of *n*-6 compared to other (e.g., *n*-3) polyunsaturated fatty acids (PUFAs) has increased considerably in the United States during recent decades. This increase in *n*-6 PUFA intake is attributed to the prevalence in modern diets of vegetable oils rich in linoleic acid (LA) [1]. The rise in PUFA consumption is consistent with recommendations to reduce intake of saturated fatty acids (SFAs) [2,3]. This is in spite of evidence about the potential intestinal proinflammatory [4,5] and carcinogenic [6] properties of diets rich in *n*-6 PUFAs. Consuming a diet high in *n*-6 PUFAs increases the risk of obesity [7], which further increases the risk of colon cancer [8] and liver steatosis [9]. Pathologies of the liver and colon are both associated with dysregulation of bile acid (BA) metabolism [10,11,12].

Bile acids are synthesized in the liver from the catabolism of cholesterol, in which cytochrome P4507A1 (CYP7A1) catalyzes the first and rate-limiting step of this process [13]. In humans, primary BA include cholic (CA) and chenodeoxycholic (CDCA) acid. From CDCA, mouse hepatic cells produce α-muricholic acid (αMCA) and its most abundant epimer βMCA, and ursodeoxycholic acid (UDCA) via 7-keto-LCA [14,15,16]. In humans, primary BA are glycine (G) (mostly) and taurine (T)conjugated (mostly taurine in rodents) and then stored in the gallbladder awaiting intestinal release upon consumption of a meal. Due to their amphipathic structure, BA are utilized in the intestinal lumen to emulsify and promote the absorption of dietary fatty acids. In the distal ileum, ~95% of conjugated BA are passively and actively absorbed and recycled back to the liver via the hepatic portal vein [17]. In the colon, primary BA that escape reabsorption (~5%) are deconjugated and 7α-dehydroxylated by the microflora leading to the production of deoxycholic (DCA) and lithocholic (LCA) acid from CA and CDCA respectively, and murideoxycholic (MDCA) from α- and βMCA [18]. In the absence of bacteria (*Lactobacillus* and *Clostridium sp*.), the TβMCA and CA cannot be metabolized respectively to murideoxycholic (MDCA) [19,20] and DCA [21].

The farnesoid X receptor (FXR) is a nuclear receptor expressed mainly in the liver, small intestine, and colon [22]. In the small intestine and colon, FXR regulates BA reabsorption through activation of various factors including small heterodimer protein (SHP) and ileal bile acid-binding (IBABP). Additionally, in the intestine FXR induces the release into the circulation of fibroblast growth factor 15/19 [FGF15 (mouse)/19(human)] [23], which induces hepatic FXR expression. In the liver, FXR activates the expression of SHP which in turn represses hepatic *CYP7A1* transcription and de novo synthesis of BA [24]. In the liver, FXR is also responsible for regulating conjugation of BA and their transport to the gallbladder. The CDCA followed by CA, DCA and LCA are agonists [25,26,27], whereas TβMCA [19] and UDCA [28] are antagonists of the FXR. Given this pleiotropic role of FXR, enhancing its expression and activation may be helpful in preventing the buildup of toxic BA and against chronic conditions including obesity [29], and hepatic [12] and intestinal [30] diseases.

The first objective of this study is to compare the effects of a diet with 28% energy (28%E) from fat and low LA (2.5%E) (CNTL) with those of an isocaloric soybean oil-based diet providing 50%E from fat and high (28%E) from LA (*n*-6HFD) on BA homeostasis in a mouse model. The second objective is to study the influence of intestinal overexpression of an *Fxrα1^TG^* on CNTL diet- and *n*-6HFD-dependent regulation of BA homeostasis. We report that the overexpression of an intestinal *Fxrα1^TG^* antagonizes the *n*-6HFD-dependent accumulation of primary BA in the cecum and liver through activation in the intestine of factors involved in their reabsorption, and repression in the liver of enzymes involved in de novo BA synthesis. Findings of this study provide further insight into the significance of targeting *Fxr* expression and activity to prevent dysregulation of BA homeostasis associated with an *n*-6HFD and promote enterohepatic health.

## 2. Results

### 2.1. Fxrα1^TG^ Mice Have Increased Expression of FXR in the Small Intestine

**Figure 1:**
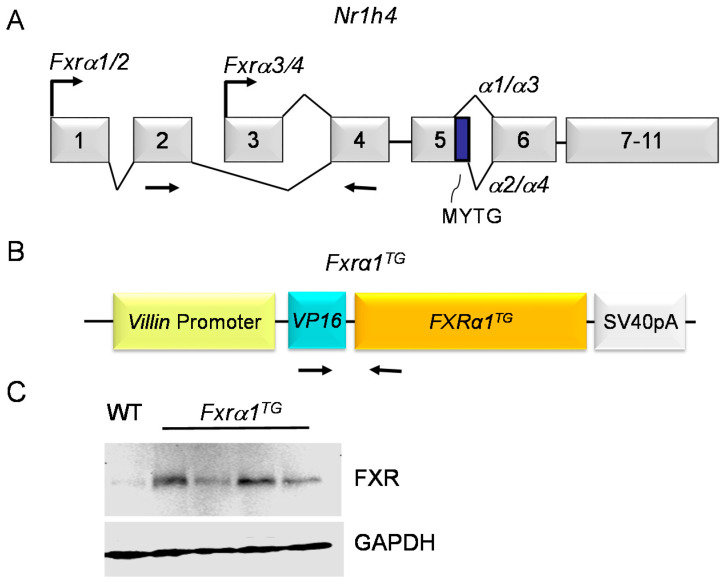
An intestinal *Villin* promoter drives expression of an *Fxrα1^TG^*. (**A**) Organization of the 11 exons of the mouse *Nr1h4* gene. Top arrows indicate alternative transcription start sites on exon 1 (α1/2 isoforms) and exon 3 (α3/4 isoforms). Top and bottom lines connecting exons indicate alternative splicing events. Bottom arrows indicate the positions of oligonucleotides used for RT-PCR. MYTG = 12 bp fragment included in the α1/α*3* isoforms. (**B**) Diagram of the *Fxrα1^TG^* construct containing a *Villin* promoter, VP16 enhancer, and a SV40polyA tail sequence. Arrows indicate the position of oligonucleotides on the *VP16* and *Fxrα1^TG^* sequences used for screening of transgenic animals. (**C**) Expression of total FXR and control GAPDH were determined by Western blot analysis in the small intestine of *Fxrα1^TG^* compared to WT littermates.

In the *Nr1h4* gene, transcription of the *Fxrα1* and *-α2* isoforms initiates on exon 1 but splices out exon 3. Further, the *Fxrα1* harbors a 12 bp nucleotide sequence encoding for a methionine-tyrosine-threonine-glycine (MYTG) insert between exon 5 and exon 6, which is spliced out in the *Fxrα2* isoform. Conversely, transcription of the *Fxrα3* and *-α4* isoforms initiates on exon 3 [31] (Figure 1A). In mice carrying the *Fxrα1^TG^* (Figure 1B) the expression of total FXR in the small intestine is increased compared to wild-type (WT) littermates (Figure 1C) providing a control for the activity of the *Fxrα1^TG^* construct in transgenic mice.

### 2.2. An n-6HFD Increases Body Weight in WT and Fxrα1^TG^ Mice

**Figure 2:**
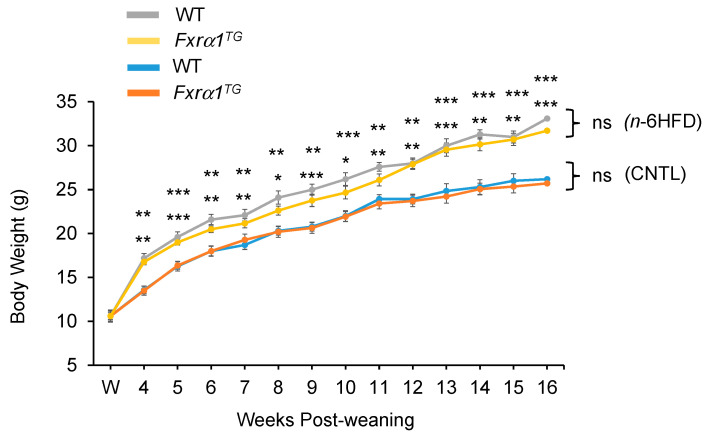
An *n*-6HFD increases body weight in WT and *Fxrα1^TG^* littermates. Data points represent sample means ± SEM from 19 individual samples. Brackets on right side of graph show no statistical difference (ns) between WT and *Fxrα1^TG^* littermates on same diet. Asterisks indicate significant difference between isocaloric CNTL-and *n*-6HFD-fed WT (top) or *n*-6HFD-fed *Fxrα1^TG^* mice (bottom) (*, *p* < 0.05; **, *p* < 0.01; ***, *p* < 0.001).

To analyze the combined effects of an *n*-6HFD and overexpression of an *Fxrα1^TG^* on end points of BA homeostasis, we fed WT and *Fxrα1^TG^* littermates a CNTL diet and an *n*-6HFD enriched with 20% soybean oil by weight (Table 1) post-weaning until 16 weeks of age. Starting at 4 weeks of age and throughout the length of this study, weekly recording of body weight indicates that the *n*-6HFD sustains greater weight gain compared to the isocaloric CNTL diet (Figure 2) in both WT and *Fxrα1^TG^* mice. There are no differences in body weight gain between the WT and *Fxrα1^TG^* littermates assigned to either dietary group.

### 2.3. n-6HFD and Fxrα1^TG^ Coordinate Enterohepatic Gene Expression

**Figure 3:**
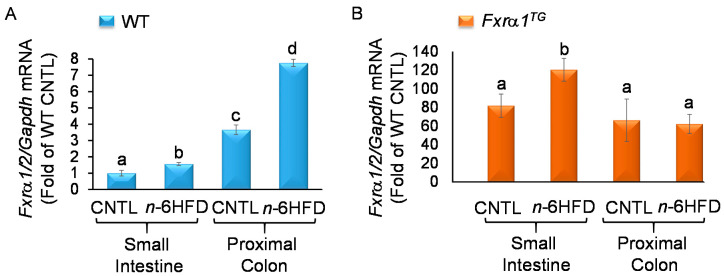
An *n*-6HFD and *Fxrα1^TG^* coordinate intestinal expression of *Fxrα1/2*. (**A**) *Fxrα1/2* mRNA expression in the small intestine and proximal colon of WT and (**B**) *Fxrα1^TG^* littermates fed an isocaloric CNTL diet or *n*-6HFD for 13 weeks. Bars represent sample means ± SEM of quantitation (fold-change of control) from five individual animals. Means without common letters (a < b < c) differ (*p* < 0.05).

Because the oligonucleotides used to amplify FXR expression flank exon 2 (forward) and exon 4 (reverse) (Figure 1A), we measured the combined expression of the *Fxrα1* and *-α2* isoforms without the confounding effects of the *Fxr-α3/4* isoforms using RT-PCR. In the small intestine of WT littermates, the *n*-6HFD increases by only ~50% (*p* < 0.05) expression of *Fxrα1/2* compared with CNTL diet (Figure 3A). In the proximal colon, however, baseline *Fxrα1/2*expression is 2.5-fold higher in CNTL mice and it increases ~6.5-fold in response to the *n*-6HFD.

Compared to WT littermates, *Fxrα1^TG^* offspring (Figure 3B) exhibit a constitutive increase in *Fxrα1/2* ranging from ~82.0 to 120-fold in the small intestine, and from ~66.0- to 62.0-fold in the proximal colon in mice fed the CNTL and *n*-6HFD, respectively. These large increases are specifically attributed to overexpression of the *Fxrα1^TG^*.

**Figure 4:**
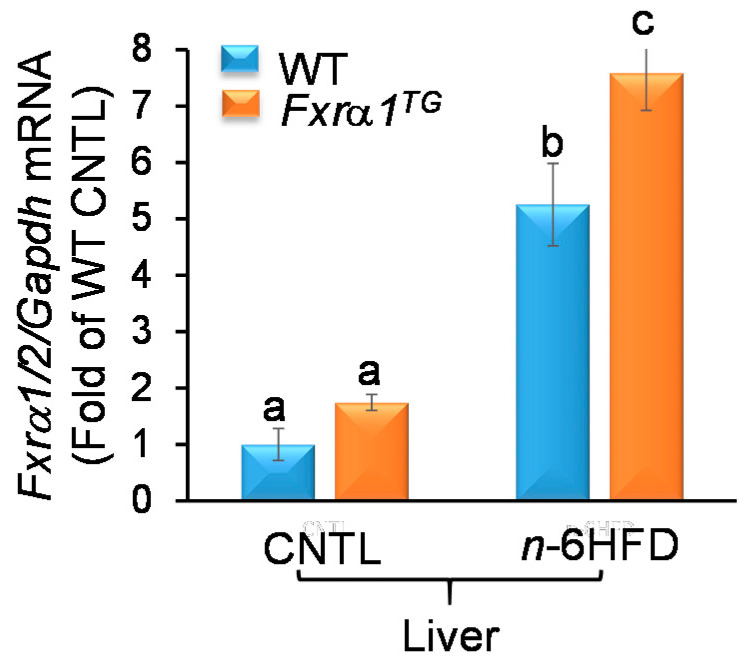
An *n*-6HFD coordinates hepatic expression of *Fxrα1/2*. *Fxrα1/2* mRNA expression in liver tissue of WT and *Fxrα1^TG^* mice on CNTL diet and *n*-6HFD. Bars represent sample means ± SEM of quantitation (fold-change of control) from five individual animals. Means without common letters (a < b < c) differ (*p* < 0.05).

In the liver, there is no difference in baseline *Fxrα1/2* expression between WT and *Fxrα1^TG^* mice on the CNTL diet (Figure 4). These expression data provide a control for intestine-specific activity of the *Villin* promoter. In contrast, hepatic *Fxrα1/2* mRNA increases significantly in response to the *n*-6HFD in WT (~4.0-fold) mice and augments further (6.5-fold) in *Fxrα1^TG^* compared to WT mice fed the CNTL diet. Overall, these data provide evidence of increased intestinal and hepatic expression of *Fxra1/2* in response to the *n*-6HFD and the *Fxrα1^TG^*.

**Figure 5:**
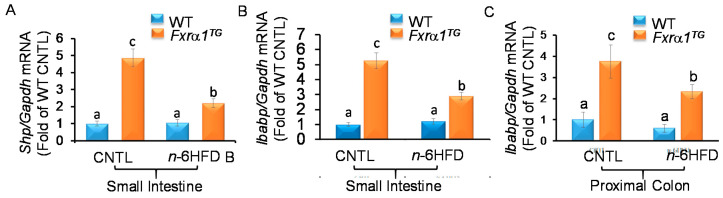
An n-6HFD antagonizes *Fxra1^TG^*-induced intestinal expression of FXR target genes in mice. (**A**) *Shp* and (**B**) *Ibabp* mRNA expression in the small intestine. (**C**) *Ibabp* mRNA expression in the proximal colon. WT and *Fxrα1^TG^* littermates were fed an isocaloric CNTL diet or an *n*-6HFD for 13 weeks. Bars represent sample means ± SEM of quantitation (fold-change of control) from five individual animals. Means without common letters (a < b < c) differ (*p* < 0.05).

An *n*-6HFD diet does not influence the expression of FXR target genes *Shp* and *Ibabp* in the small intestine (Figure 5A,B, respectively), or *Ibabp* in the proximal colon (Figure 5C). The *n*-6HFD only slightly decreases *Ibabp* mRNA in the proximal colon of WT mice. As expected, in association with the CNTL diet, the *Fxrα1^TG^* supports the accumulation in the small intestine of *Shp* and *Ibabp* (~4.0-fold), and of *Ibabp* (2.7-fold) in the proximal colon. However, compared to CNTL diet, in the *Fxra1^TG^* animals, the *n*-6HFD antagonizes the stimulation of *Shp* and *Ibabp* in the small intestine, and of *Ibabp* in the proximal colon.

**Figure 6:**
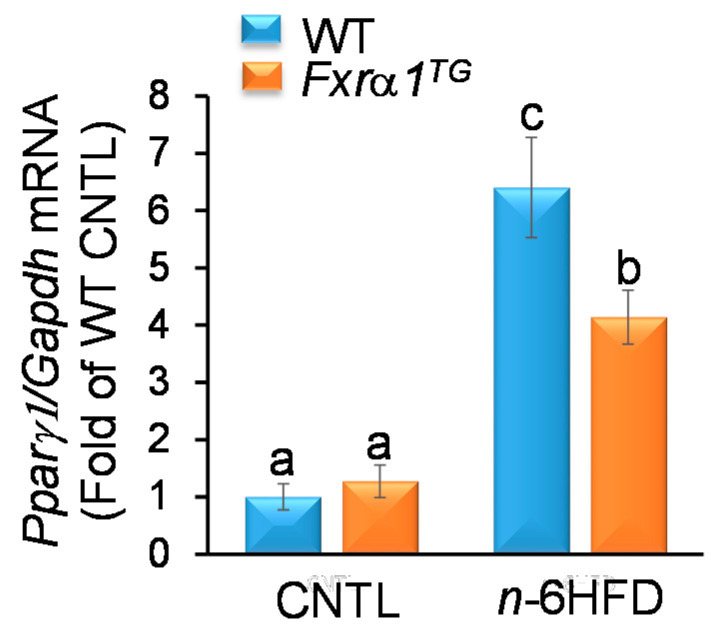
An *n*-6HFD and *Fxrα1^TG^* regulate *Pparγ1* expression in the small intestine. WT and *Fxrα1^TG^* littermates were fed an isocaloric CNTL diet or an *n*-6HFD for 13 weeks. Bars represent sample means ± SEM of *Pparγ1* mRNA quantitation (fold-change of control) from five individual animals. Means without common letters (a < b < c) differ (*p* < 0.05).

The expression of FXR impacts also on expression of factors involved in fatty acid metabolism including peroxisome-proliferator-activated receptor-γ (*Pparγ*) [32]. Previously, we [33] and others [34] have reported that a HFD increases the expression of *Pparγ1* in the small intestine. Analysis of *Pparγ1* mRNA expression in the small intestine shows no difference between WT and *Fxrα1^TG^* mice on the CNTL diet; however, the *n*-6HFD increases *Pparγ1* in WT (~6.4-fold) and *Fxrα1^TG^* littermates (~4.1-fold, Figure 6). These data provide a positive control for the effects of the *n*-6HFD on lipid metabolism in the small intestine and evidence for the attenuating effects of the *Fxrα1^TG^*.

Overall, these expression data indicate that the *Fxrα1^TG^* leads to activation of downstream targets for the FXR in the small intestine (i.e., *Shp*, *Ibabp*) and proximal colon (*Ibabp*) on the CNTL diet. These effects are hampered by an *n*-6HFD. Moreover, the feeding of an *n*-6HFD enhances hepatic *Fxrα1/2* expression, which is amplified in association with the transgenic overexpression of *Fxrα1^TG^* in the intestine.

### 2.4. An n-6HFD and Fxrα1^TG^ Coordinate Enterohepatic Ba Homeostasis

**Figure 7:**
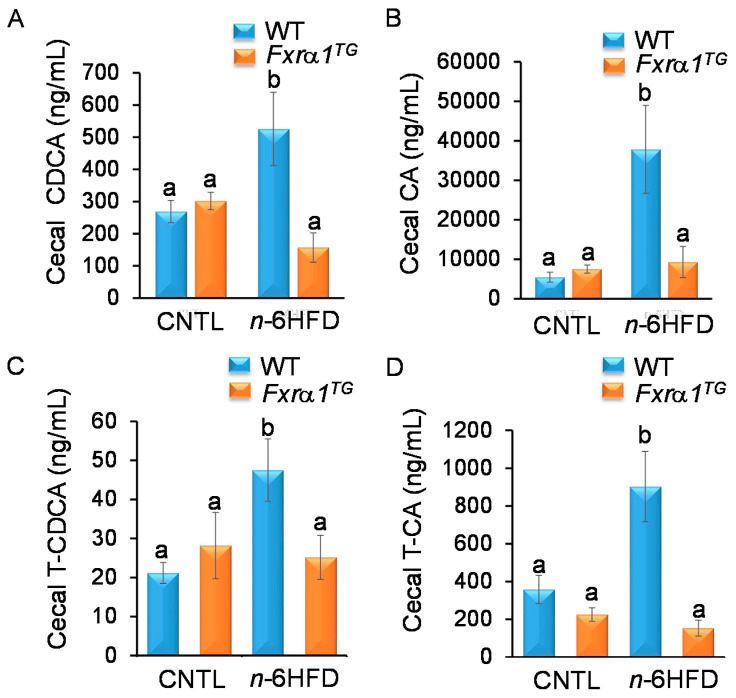
An *n*-6HFD-mediated increase in cecal levels of primary and conjugated BA is attenuated by an *Fxrα1^TG^*. Concentration of (**A**) CDCA, (**B**) CA, (**C**) T-CDCA, and (**D**) T-CA (ng/mL) from cecal contents. Bars represent sample means ± SEM from seven individual animals fed an isocaloric CNTL diet or an *n*-6HFD for 13 weeks. Means without common letter (a < b < c) differ (*p* < 0.05).

To detail the impact of the *n*-6HFD and *Fxrα1^TG^* alone or in combination on regulation of BA homeostasis, we measured BA levels in cecal pellets and hepatic tissue. Results of HPLC/MS depicted in Figure 7A indicate that the cecal concentration of the primary CDCA and CA is not different between WT and *Fxrα1^TG^* littermates on the CNTL diet. Conversely, the *n*-6HFD increases the cecal levels of CDCA and CA (Figure 7B). Interestingly, cecal CDCA and CA are reduced to CNTL levels in *Fxrα1^TG^* mice (Figure 7A,B). Because bile acids in mice are conjugated predominantly with the amino acid taurine, we measured the levels of taurine-CDCA (T-CDCA) and -CA (T-CA). Similar to unconjugated CDCA and CA, levels of T-CDCA (Figure 7C) and T-CA (Figure 7D) are increased in cecal pellets from mice fed the *n*-6HFD, and this effect is antagonized by expression of the *Fxrα1^TG^*.

These results suggest that intestinal expression of the *Fxrα1^TG^* attenuates the levels of primary and conjugated BA that escape reabsorption in the small intestine as a result of feeding the *n*-6HFD.

**Figure 8:**
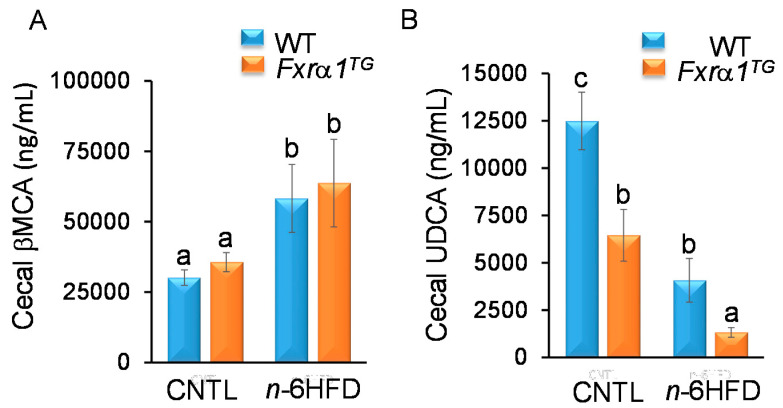
Effects of *n*-6HFD and *Fxrα1^TG^* on cecal levels of primary βMCA and UDCA. Bars represent mean concentration of (**A**) βMCA and (**B**) UDCA (ng/mL) from cecal samples ± SEM from seven individual animals fed an isocaloric CNTL diet or an *n*-6HFD for 13 weeks. Means without common letter (a < b < c) differ (*p* < 0.05).

In mice, both βMCA and UDCA are considered as primary BA synthesized from CDCA [19]. Our results (Figure 8A) show that the cecal concentration of βMCA exceeds by ~100-fold that of CDCA and is not different in mice fed the CNTL diet regardless of genotype (i.e., WT vs. *Fxrα1^TG^*). These results are in agreement with the notion that in mice most CDCA is transformed in the liver into βMCA [15]. However, the concentration of βMCA nearly doubles in cecal pellets from mice fed the *n*-6HFD. In contrast to what seen for primary CDCA and CA, the expression of the *Fxra1^TG^* does not impact on the cecal levels of βMCA. Conversely, levels of UDCA in cecum are reduced by the *Fxrα1^TG^* compared to WT littermates on the CNTL diet, and to a larger degree in association with the *n*-6HFD (Figure 8B).

**Figure 9:**
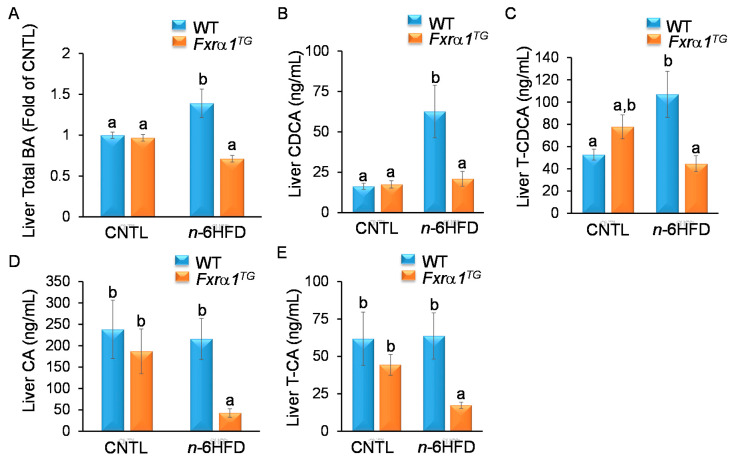
An *n*-6HFD-mediated increase in hepatic primary BA is attenuated by an *Fxrα1^TG^*. (**A**) Total BA. (**B**) CDCA. (**C**) T-CDCA. (**D**) CA). (**E**) T-CA. Bars represent sample means ± SEM from seven individual animals fed an isocaloric CNTL diet or an *n*-6HFD for 13 weeks. Means without common letter (a < b < c) differ (*p* < 0.05).

Analysis of liver homogenates suggests no difference in the concentration of total BA between WT and *Fxrα1^TG^* mice on the CNTL diet (Figure 9A). On the other hand, WT animals fed the *n*-6HFD exhibit an increase (~40%) in total BA compared to CNTL-fed mice, which are lowered to near CNTL levels in *Fxrα1^TG^* mice. These data suggest that overexpression of the *Fxrα1^TG^* in the intestine attenuates the *n*-6HFD-mediated increase in total BA in the liver.

**Figure 10:**
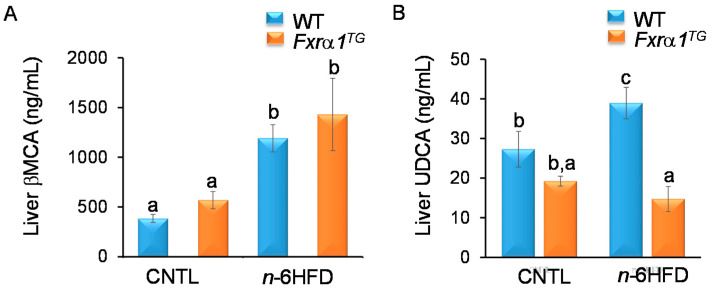
Effects of *Fxrα1^TG^* on hepatic concentrations of (**A**) *β*MCA and (**B**) UDCA. Bars represent sample means ± SEM from seven individual animals fed an isocaloric CNTL diet or an *n*-6HFD for 13 weeks. Means without common letter (a < b < c) differ (*p* < 0.05).

Similarly, the liver concentration of CDCA shows no difference between WT and *Fxrα1^TG^* littermates on the CNTL diet; however, treatment with the *n*-6HFD increases the concentrations of CDCA (Figure 9B) and T-CDCA (Figure 9C) in WT mice, which are attenuated in *Fxrα1^TG^* littermates (Figure 9B,C). We can see that in transgenic mice on the CNTL diet, the CA and T-CA are lower compared to WT mice (Figure 9D,E). These data support the notion that *Fxra1/2* negatively affects CA and T-CA in the liver, and this repression is augmented by *n*-6HFD diet, as expected, since *n*-6HFD induces overexpression of *Fxra1/2* in the small intestine (Figure 3B). The concentration of βMCA in the liver mirrors that of the cecum, increasing with the *n*-6HFD, with no effects due to genotype (Figure 10A). 

For UDCA, higher levels are seen in WT mice compared to *Fxrα1^TG^* littermates fed either the CNTL or *n*-6HFD diet (Figure 10B). Taken together, these data suggest that an *n*-6HFD increases the levels of total BA, and primary CDCA and T-CDCA in the liver, and that the intestinal overexpression of the *Fxrα1^TG^* attenuates these effects. However, the *Fxrα1^TG^* does not impact on the hepatic concentration of murine-specific βMCA irrespective of type of diet.

**Figure 11:**
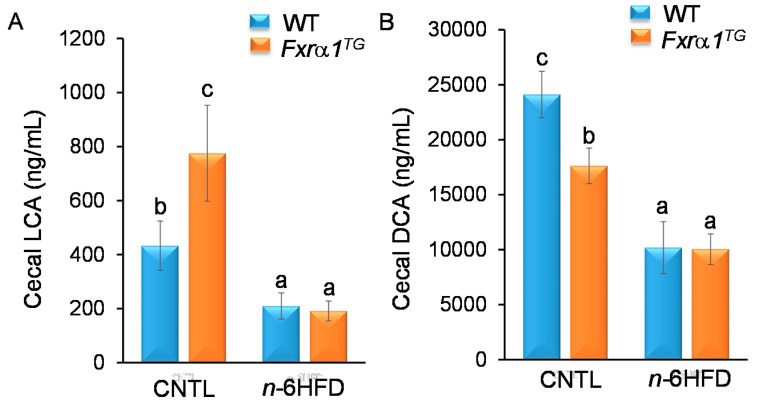
An *n*-6HFD reduces the cecal levels of secondary LCA and DCA. Concentration (ng/mL) of (**A**) LCA and (**B**) DCA in cecal contents. Bars represent sample means ± SEM from seven individual animals fed an isocaloric CNTL diet or an *n*-6HFD for 13 weeks. Means without common letter (a < b < c) differ (*p* < 0.05).

We then measured the cecal levels of DCA and LCA because in humans they represent the majority of secondary BA. The concentration of LCA is considerably lower (~20-fold) (Figure 11A) compared to that of DCA (Figure 11B) likely due to the fact most CDCA, the precursor for the synthesis of LCA, is transformed in mice into βMCA. The cecal concentration of DCA is about 2.5 times higher than that of CA and similar to βMCA, in contrast with the 4:4:2 CA/CDCA/DCA distribution generally seen in humans [35]. Levels of LCA are increased in *Fxrα1^TG^* mice on the CNTL diet; however, *Fxrα1* overexpression has a negative effect on cecal DCA in CNTL diet mice, whereas the *n*-6HFD negatively influences LCA and DCA levels in both WT and transgenic mice. Taken together, these data suggest a differential effect between *n*-6HFD and *Fxrα1^TG^* overexpression on the relative production of secondary LCA and DCA in the cecum.

### 2.5. An n-6HFD and Fxrα1^TG^ Regulate Expression of Genes Involved in BA Homeostasis

**Figure 12:**
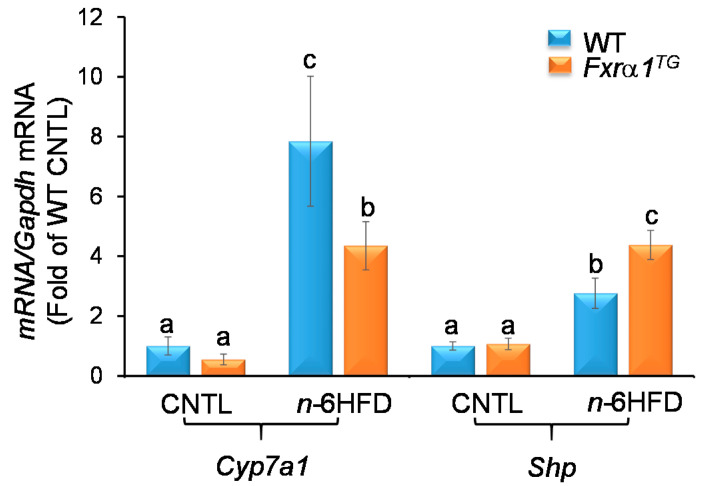
An intestinal *Fxrα1^TG^* attenuates *n*-6HFD-associated hepatic expression of *Cyp7a1* via upregulation of *Shp*. Bars represent means ± SEM of *Cyp7a1* and *Shp* mRNA in hepatic tissue of WT and *Fxrα1^TG^* littermates fed an isocaloric CNTL or *n*-6HFD for 13 weeks. Quantitation (fold-change of control) from five individual animals. Means without common letter (a < b < c) differ (*p* < 0.05).

The activation of FXR in the intestine represses the de novo synthesis of BA in the liver through a feedback loop [22]. In the small intestine, FXR induces the expression of FGF15, which signals back to the liver to activate FXR. The latter induces the hepatic expression of SHP that prevents liver related homolog-1 (LRH-1) and hepatocyte nuclear factor 4α (HNF4α) from promoting the transcription of Cyp7a1 [36]. The CYP7A1 enzyme participates in the de novo synthesis of BAs. Our results show that the n-6HFD triggers an increase in both hepatic Cyp7a1 and Shp mRNA expression (Figure 12). However, intestinal overexpression of Fxra1^TG^ does not affect Shp levels in mice fed the CNTL diet, but attenuates Cyp7a1 expression in association with n-6HFD diet. In line with these observations, hepatic Shp expression is upregulated ~2.8- and ~4.4-fold respectively in WT and Fxrα1^TG^ mice on the n-6HFD.

In summary, these data suggest that intestinal overexpression of Fxrα1 reduces the amounts of primary BA that escape reabsorption, and triggers a negative feedback response in the liver mediated by FXR via SHP leading to suppression of Cyp7a1 expression thus attenuating de novo synthesis and the consequent accumulation of BA.

#### Summary

Overall, the results illustrated in this study indicate that an n-6HFD (~50%E from fat) increases body weight (Figure 2) and affects BA metabolism. Key findings of this study are:

1. An *n*-6HFD diet moderately affects *Fxrα1/2* expression in the small intestine (Figure 3A) and to a larger extent in the proximal colon (Figure 3A) and liver (Figure 4).
2. Compared to a CNTL diet (~28%E from fat)*,* an *n*-6HFD diet does not influence the expression of FXR target genes *Shp* and *Ibabp* in the small intestine (Figure 5A,B), or *Ibabp* in the proximal colon (Figure 5C).
3. An *n*-6HFD diet induces hepatic *Cyp7a1 and Shp* expression (Figure 12), which may contribute to the de novo synthesis of BAs (Figure 9A–C).
4. An *n*-6HFD diet augments *Pparγ1* expression in the small intestine, supporting its role on fatty acid metabolism (Figure 6).
5. An *n*-6HFD diet increases cecal CDCA and CA levels (Figure 7), and liver total BAs.
6. An *n*-6HFD reduces cecal secondary BAs (LCA, DCA) independently of *Fxrα1* transgene expression (Figure 11).
7. Intestinal *Fxrα1* transgene overexpression (Figure 1) induces expression of FXR target *Shp* gene (Figure 5A–C) in the small intestine and colon, as expected, particularly under CNLT diet.
8. Intestinal *Fxrα1* overexpression, either under the CNTL or *n*-6HFD diet, does not seem to affect cecal CDCA or CA levels (Figure 7A,B) or liver total BA concentration (Figure 9A), particularly CDCA (Figure 9B).

## 3. Discussion

Bile acids are necessary for intestinal emulsification and absorption of dietary fatty acids. However, excessive BA accumulation in the intestine and liver promotes inflammation and tissue damage [10,11,12]. The nuclear receptor, FXR, regulates BA homeostasis throughout the enterohepatic system [22]. The present study addresses the effects of an *n*-6HFD enriched in LA on intestinal and hepatic expression of genes involved in BA metabolism, and the modifying role of intestinal *FXRα1^TG^* expression on enterohepatic BA homeostasis.

In modern diets, *n*-6 LA is described as the primary dietary PUFA [37]. However, the increased intake of *n*-6 PUFAs at the expense of other fatty acids (i.e., *n*-3) increases the risk of chronic diseases such as obesity, and cardiovascular and nonalcoholic fatty liver disease (NAFLD) [38]. Additionally, higher consumption of *n*-6 PUFAs increases the risk of intestinal inflammation [4,5,33] and promotes the development of colon cancer [39,40,41,42]. A contributing factor to these conditions is dysregulation of BA homeostasis. Therefore, in order to model the effects of a dietary pattern rich in *n*-6 on end points of BA metabolism, we examined the effects of a *n*-6HFD providing ~50%E from total fat, of which ~28%E is from *n*-6 LA, with those of a CNTL palm oil-based containing ~28%E from total fat, of which only ~2.5%E is from LA. In accord with previous work by our group [33] and other investigators [34,39,43] with similar *n*-6 LA-enriched diets, the current study shows that an *n*-6HFD triggers higher weight gain compared to an isocaloric CNTL diet with a lower fat content.

In the background of control WT mice, the expression of Fxrα1/2 is higher in the proximal colon compared to that of the small intestine and this difference in amplified by the *n*-6HFD. One possible explanation for this differential expression may relate to tissue-specific regulation of *Fxra1/2*. For example, transcripts for the *Fxrα1/2* isoforms are nearly undetectable in the duodenum but increase in the jejunum and even more in the ileum [31]. Possibly, this expression gradient may extend to the proximal colon since higher expression of FXR in the colon compared to the small intestine has been previously documented in mice [44]. The *n*-6HFD also increases the expression of endogenous *Fxrα1/2* in the liver. These expression changes likely represent adaptive intestinal and hepatic responses to higher intake of fat [22,45]. In support of this interpretation, we observe in the cecal material an increase in unconjugated (CDCA, CA) and conjugated (T-CDCA,T-CA), and murine-specific βMCA, primary BA. These changes are mirrored in the liver by accumulation of total BA, CDCA, βMCA, and UDCA, and increased expression of *Cyp7a1* and *Shp*. The CYP7A1 enzyme catalyzes in the liver the first and rate-limiting step of BA synthesis, converting cholesterol to primary CDCA and CA [46]. Therefore, the *n*-6HFD sustains de novo hepatic BA synthesis, which correlates with higher risk of developing NAFLD [12], nonalcoholic steatohepatitis [47], and hepatic cancer [48].

The *NR1H4* gene encodes four isoforms (FXRα1, -α2, α3, and -α4), which result from tissue-specific alternative transcription and splicing [49]. Both FXRα1 and FXRα2 are expressed at comparable levels in the intestine of mice. Additionally, analysis of murine *Fxr* gene isoform expression and function shows that FXRα1 and FXRα2 activate transcription of *Shp* and bile salt export pump to a similar extent [31,50]. Considering the role of FXR in regulating BA metabolism, we developed a transgenic mouse model overexpressing murine *Fxrα1* under the control of an intestine-specific *Villin* promoter. The *Fxrα1^TG^* drives increased expression of *Shp* and *Ibabp*, two downstream targets for FXR [51,52]. However, these stimulatory effects on *Shp* and *Ibabp* are hampered in the small intestine and proximal colon by the *n*-6HFD possibly through several, non-mutually exclusive, mechanisms. First, the repression on *ShP* and *Ibabp* may occur independent of FXR through transcription factors as has been shown for the vitamin D receptor (VDR) which represses transcription through binding at VDR elements located within the proximal *Shp* promoter [53]. The VDR can be activated by the secondary LCA [54], whose cecal levels however were reduced by the *n*-6HFD. Second, the accumulation of βMCA, a known antagonist of the FXR [27] may prevent FXR-dependent activation of *Shp* and *Ibabp*, in spite of increased FXR levels. Third, the reduction in expression of *Shp* and *Ibabp* associated with the *n*-6HFD may be related to microbial inhibition of FXR signaling [20,55]. In support of this mechanism, studies have shown that the administration to C57BL/6J male mice of bacterial metabolites from *Eubacterium Limosum* in combination with an HFD (10 weeks starting at week 5 of age) activates *Fxr* expression, but opposite to bacterial metabolites from *Bacteroides dorei,* repress ileal expression of *Shp* [56]. Therefore, changes in the gut microbia due to an n-6HFD may alter the relative balance in the intestine in favor of FXR-inhibitory BAs and metabolites.

In mice fed the *n*-6HFD, the intestinal overexpression of the *Fxrα1^TG^* mitigates the increase in total and primary hepatic CDCA and CA observed in WT littermates suggesting that the *Fxr^TG^* promotes intestinal primary BA reabsorption. In the enterocyte, FXR induces the expression and subsequent release into the circulation of FGF15, which in turn inhibits hepatic *Cyp7a1* [23,57]. In fact, the hepatic expression of *Cyp7a1* is attenuated in *Fxrα1^TG^* mice fed the *n*-6HFD suggesting the *Fxrα1^TG^* is signaling back to the liver to suppress de novo BA synthesis via repression of *Cyp7a1*. In support of this interpretation, we show that in addition to intestinal overexpression of *Fxrα1*, transgenic animals on the *n*-6HFD exhibit higher expression of hepatic *Fxrα1/2* mRNA. Similarly, other studies reported increased expression of hepatic *Fxrα* in response to an HFD [45,58]. Mechanistically, the activation of FXR in the liver drives expression of SHP, which in turn inhibits the transcription of *Cyp7a1* via interactions with LRH-1 and HNF4α [36,59]. Therefore, the combined increase in intestinal and hepatic FXR expression seen in Fxrα*1^TG^* littermates operate respectively to increased reabsorption and reduce de novo synthesis.

The PPARγ participates in regulation of lipid and glucose metabolism. Natural and synthetic ligands of the FXR induce PPARγ expression [60]. Activation of FXR by CDCA increases transcription of *PPARγ* via binding to an FXR-responsive element (FXRE) harbored in the *PPARγ* promoter [32]. Similar to humans, the mouse *Pparγ* gene encodes for two isoforms (*Pparγ1* and *Pparγ2*), of which *Pparγ1* is expressed at higher levels in adipose tissue and intestine [61]. In keeping with the role of PPARγ in lipid metabolism, we find that in WT littermates the intestinal expression of *Pparγ*1 is induced by the *n*-6HFD in association with accumulation of CDCA and upregulation of endogenous *Fxrα1/2*. On the other hand, *Pparγ1* expression is hampered in the small intestine of *Fxrα1^TG^* mice fed the *n*-6HFD. This reduction may result from lower transactivation activity by the overexpressed *Fxrα1* isoform at the *Pparγ1* FXRE and other FXR target genes [62] compared to other endogenous FXR isoforms (i.e., *FXRα2*) [31]. Another factor that may contribute to reducing *Pparγ* expression in the intestine is the FXR–FGF15/19 axis. Although in the liver this mechanism has been demonstrated for the *Pparγ2* isoform [63], it remains unknown whether it is operative for *Pparγ1* in the intestine.

Interestingly, overexpression of the *Fxrα1^TG^* does not affect the *n*-6HFD-associated accumulation of unconjugated βMCA in cecal samples and liver. Further, the levels of secondary LCA and DCA in cecal samples are markedly reduced by the *n*-6HFD in both WT and *Fxrα1^TG^* littermates. A possible interpretation of these data is that the *n*-6HFD influences on the microbiota and FXR signaling. For example, studies show that *n*-6LA is toxic to probiotic *Lactobacillus* [64,65,66]. A feeding study also shows that there is an increase in *Bacteroidetes* and a dramatic reduction in *Firmicutes* in association with a diet rich in LA (safflower oil, 37%E), compared to a low-fat group and an isocaloric lard group [67]. *Firmicutes* is a bacterial phylum that includes *Lactobacillus* and *Clostridium*. The bacteria that produce DCA from CA belong to the genus *Clostridium*. The lowering of the genera *Lactobacillus* and *Clostridium* increases the levels of conjugated βMCA, which with DCA acts as an antagonist on FXR [19,30], and reduces FXR signaling [18,20]. Therefore, the accumulation of βMCA may explain at least in part the lower expression of *Shp* and *Ibabp* noted in the small intestine and proximal colon of *Fxrα1^TG^* littermates fed the *n*-6HFD. Conversely, other studies show an increase in *Clostridium* in animals fed a high-fat palm oil (45%E) diet compared to safflower oil (45%E), olive oil, and low-fat diets [34]. Βased on this background, a diet rich in LA may be toxic to *Lactobacillus* and *Clostridium* population in the gut and ameliorate the signaling effects of FXR.

## 4. Materials and Methods

### 4.1. Animals

**Figure 13:**
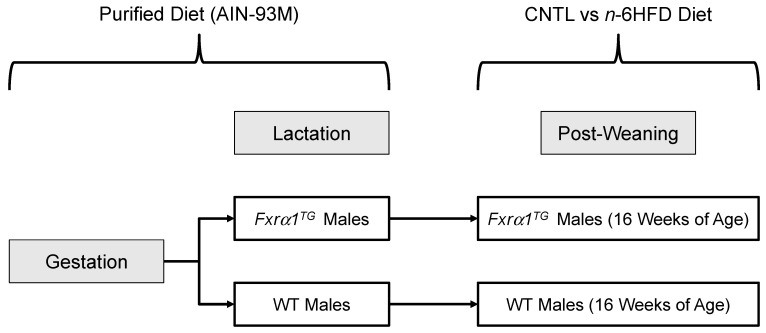
Schematics describing experimental design of feeding study. Breeder pairs were fed the AIN-93M Purified Diet throughout gestation and lactation. After weaning, WT and *Fxrα1^TG^* male littermates were assigned to CNTL or *n*-6HFD for 13 weeks until 16 weeks of age.

Transgenic *Fxrα1* were generated using C57BL/6J zygotes and were subsequently crossed with C57BL/6J mice for several generations until there was stable transmission of the same copy number of the *Fxrα1^TG^*. Two founders, 1R and 3, contained 1 and 2 copies of the transgene, respectively, and produced mice with intestinal overexpression of Fxrα1. Animals from founder 3 were used for the experiments presented here. Oligonucleotides for identification of VP16 positive transgenic mice were, forward: 5′-TGGGCCCTAAAAAGAAGCGT-3′; reverse: 5′-ATCGAAATCGTCTAGCGCGT-3′. Breeder pairs were assigned to AIN93M Purified Diet (Table 1). Weaned WT and Fxrα1^TG^ mice at 3 weeks of age were assigned to a CNTL diet containing 27.8%E from fat (11% palm oil by weight) (Figure 13) or an n-6HFD containing 50.3%E from fat (20% soybean oil by weight), until the end of this study (16 weeks of age). The relative energy contribution by SFAs, MUFAs, and PUFAs was 7%, 12%, and 31% for the *n*-6HFD diet, and ~14%, 11.0%, and 3% for the CNTL diet, respectively. Litters were allowed chow and water ad libitum, and weights were measured weekly.

At the end of the 16 week experimental period, animals were sacrificed and liver samples, cecal pellets, and mucosa from the small intestine and proximal colon were collected (Figure 13). The collection of the mucosal cells was performed as previously described [68]. The small intestine and colon were cut longitudinally then rinsed with phosphate buffered saline (PBS) and scraped. The scraped cells were then separated after centrifugation for 10 min at 4 °C. All animal procedures were approved by the Institutional Animal Care and Use Committee program of the University of Arizona (PHS Animal Welfare Assurance Number D16-00159, A3248-01, effective 08-08-2019).

### 4.2. mRNA Analysis

Preparation of mRNA from small intestine and proximal colon mucosal cells and liver tissue was performed using the Quick-RNA MiniPrep kit as per the manufacturer’s instructions (Zymo, Irvine, CA, USA; Ref. 11-328). Briefly, specimens were suspended in RNA lysis buffer and sonicated on ice for 4 pulses of 10 s each. DNA was digested using DNase I and RNA was eluted using RNase-free water. Purified RNA was stored at −80 °C or used immediately for cDNA synthesis using the qScript cDNA Synthesis Kit as per the manufacturer’s instructions (Quantabio, Beverly, MA, USA; Ref. 95047-025). Purified cDNA was stored at −20 °C or used immediately in real-time qPCR assays carried out in a 20 μL volume with a master mix consisting of 10 μL of PerfeCta SYBR Green FastMix with carboxyrhodamine (ROX) (Quantabio), 2 μL of 5 μM forward and reverse primers, 4 μL of RNase-free water, and 2 μL of cDNA template. Reaction parameters for PCR were: 95 °C for 10 min (escalating by 1.6 °C/s), followed by 40 cycles of 95 °C for 15 s, with an annealing temperature of 65 °C for 1 min. Relative mRNA quantities were determined using the relative standard curve method [69] using GAPDH as an internal standard. Mouse primer sequences (Sigma Aldrich, St. Louis, MO, USA) are shown in Table 2. Primers for *Fxrα* expression designate *Fxrα1* and *Fxrα2* expression combined, but exclude *Fxrα3* and *Fxrα4* (Figure 1).

### 4.3. Western Blot Analysis

Western blotting was performed as previously described [33]. Briefly, total protein was extracted from colonic mucosa by suspending ~30 mg of tissue in Pierce RIPA Buffer (Thermo Fisher Scientific, Waltham, MA, USA) containing a 1% concentration of protease inhibitor (VWR, Ref. M250). Samples were incubated on ice for ~45 min with occasional vortexing. After incubation, samples were centrifuged at 16,000× *g* for 10′ at 4 °C to separate cell debris from the protein lysate. Protein concentration was determined using the Nanodrop1000 Spectrophotometer (Thermo Fisher Scientific, Waltham, MA, USA). Samples were prepared for polyacrylamide gel electrophoresis (PAGE) by heating 100 μg of protein (normalized with water) at 65 °C for 4 min. Following this heating step, an equal volume of Leamlli buffer (Biorad, Hercules, CA, USA; Ref. 161-0737) containing 1% β-mercaptoethanol was added. This mixture was boiled in a hot water bath for 4 min, cooled to room temperature for 4 min, then centrifuged at 10,000× *g* for 30 s. Proteins were separated on Novex Wedgewell 4–12% tris-glycine gels (Invitrogen, Carlsbad, CA, USA; Ref. XP04120BOX) using a constant voltage (100 V) for ~75 min. Proteins were transferred to nitrocellulose membranes (Amersham, Little Chalfont, UK; 10600001) using the Invitrogen Mini Blot Module (B1000) and Mini Gel Tank (A25977) wet-transfer system. Transfer was conducted in tris-glycine transfer buffer (15% methanol) at 15 V for 45 min. Blocking was performed for 1 h at room temperature with a 1% casein blocking buffer dissolved in tris-buffered saline containing 1% NaCl. Immunoblotting was carried out using the primary antibody FXR(C-20):SC-1204 and GAPDH (Santa Cruz Biotechnology, Dallas, TX, USA) and secondary antibodies specific to rabbit (Li-COR, Lincoln, NE, USA). Antibodies were diluted in 1% casein blocking buffer dissolved in TBS + 0.01% tween (TBS-T) and primary incubations were carried out overnight at 4 °C. Following primary incubation, membranes were incubated in secondary antibody for 1 h at room temperature. Immunocomplexes were detected by near-infrared scanning using an Odyssey CLx (Li-COR, Lincoln, NE, USA). Quantitation was performed using ImageStudio Lite.

### 4.4. Total Bile Acids

Total BA determination was carried out using the Diazyme Total Bile Assay kit according to manufacturer instructions (Diazyme Laboratories Inc. cat # DZ042A-K01, Poway, CA, USA). Briefly, total BA were extracted from ~30 mg of cecal pellet or liver tissue as follows: after weight measurement, 4 volumes of extraction buffer consisting of 95% ethanol and 0.1 M NaOH were added to each sample which was then homogenized using a hand-held homogenizer (BelArt H-B Instrument, SP Scienceware, Wayne, NJ, USA). Samples were incubated at 80 °C for 1 h, centrifuged at 12,000 rpm for 15 min and 3 μL of the supernatant were used for the assay. The total BA assay was conducted in triplicate for each sample, readings were carried out at 405 nm using a Synergy HT 96 well plate reader using a KC4 software (Bio-teck, Orlando, FL, USA). Concentrations were calculated using BA standards (Diazyme).

### 4.5. Liquid Chromatography and Mass Spectrometry

Cecal pellets and liver tissues were homogenized in 4 volumes of extraction buffer made up of 95% ethanol and 0.1 M NaOH. Free and conjugated BA were extracted from samples as previously described [70]. Briefly, separation was completed with the use of a gradient system of acetonitrile, water and 0.1% formic acid. The detection of BA from the samples was completed as previously described [71]. The detection of free and conjugated BA was completed by measuring negative ions.

### 4.6. Statistical Analysis

Differences between groups were analyzed by two-way ANOVA using a mixed model to correct for differences in group size. Significant differences (*p* < 0.05) were determined using Tukey’s HSD test. Statistical analysis was performed using Prism (Graph-Pad Software, San Diego, CA, USA).

## 5. Conclusions

**Figure 14:**
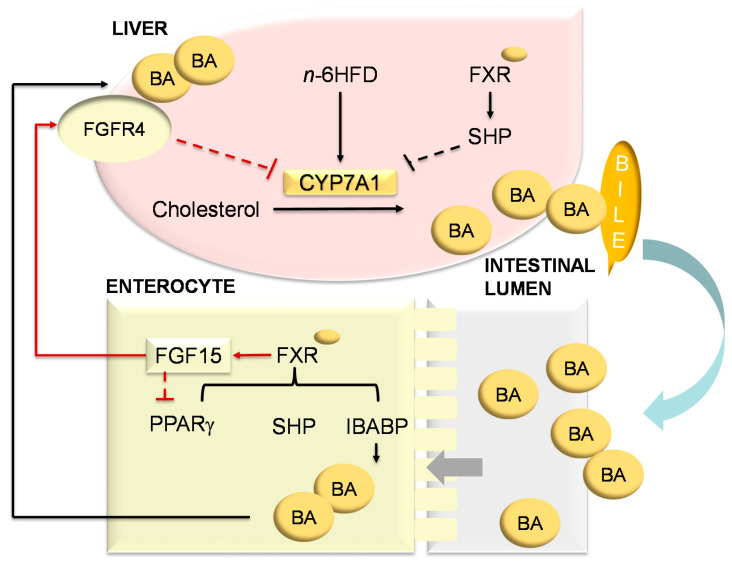
Proposed effects of an *n*-6HFD on BA metabolism and the impact of intestinal overexpression of *Fxr*. The long-term exposure to an *n*-6HFD increases hepatic *Cyp7a1* and BA accumulation in the intestine and liver. These effects are attenuated in *Fxra1^TG^* mice. Activation of FXR in the intestine promotes expression of FGF15, which activates a signaling cascade in the liver through the FGFR4 to inhibit *Cyp7a1* expression [23,74]. Black arrows denote relationships supported by the current data. Red arrows depict relationships characterized previously [22,75].

Results from the current study are illustrated in Figure 14 showing that the long-term exposure to an *n*-6HFD promotes de novo synthesis of BA associated with increased expression of *Cyp7a1* and higher levels of primary BA in the cecum in male mice. Conversely, an *Fxra1^TG^* hampers hepatic *Cyp7a1* gene expression and reduces the concentration of primary BA in the cecum and liver tissues. These observations suggest that the *Fxra1^TG^* transgenic model may be useful to elucidate the role of intestinal FXR expression and activation for the prevention of enterohepatic diseases. Although this study does not focus on cancer end points, we have shown that inactivation of the adenomatous polyposis Coli (APC) predisposes to epigenetic silencing of FXR in the colon in previous studies [72]. Because ~70% of colorectal cancers have CpG hypermethylated *APC*, the *Fxrα1^TG^* mouse model may be useful for studies of colorectal cancer prevention and treatment linked to HFD and silencing of *APC*. Finally, future studies should extend these investigations to the female gender for which differences in BA and microbiota related to diet and FXR have been documented [73].

## Figures and Tables

**Table 1:** Diet Composition. ^a^

Diet Formula	AIN-93M Purified Diet (g/kg)	CNTL (g/kg)			*n*-6HFD (g/kg)	
Casein	140.0	140.0			140.0	
L-Cystine	1.8	1.8			1.8	
Corn Starch	465.7	267.5			87.5	
Maltodextrin	155.0	155.0			155.0	
Sucrose	100.0	100.0			100.0	
Soybean Oil	40.0				200.0	
Palm Oil		110.0				
Cellulose	50.0	155.0			290.0	
Mineral Mix, AIN-93M-MX (94049)	35.0	35.0			35.0	
Mineral Mix, AIN-93-VX (94047)	10.0	10.0			10.0	
Choline Bitartrate	2.5	2.5			2.5	
TBHQ, Antioxidant	0.01	0.02			0.04	
Nutrient Composition	% Weight	% Kcal	% Weight	% Kcal	% Weight	% Kcal
Protein	12.4	13.7	12.4	13.7	12.4	13.7
Carbohydrate	68.3	75.9	52.8	58.4	32.3	35.9
Fat	4.1	10.3	11.1	27.8	20.1	50.3
Energy (Kcal/g)	3.6	3.6			3.6	

^a^ values are calculated from ingredient analysis or manufacturer data (Teklad Laboratory). *n*-6HFD = diet enriched with n-6 fatty acids; TBHQ = tertiary butyl-hydroquinone. CNTL diet contains 27.8% E from fat (11% palm oil by weight); n-6HFD contains 50.3% E from fat (20% soybean oil by weight)

**Table 2:** RT-PCR Oligonucleotide Sequences.

Target	Primer Sequence
*Fxrα1/2*	F: 5′-GGCTACGGACGAGTTTTCTCT-3′
	R: 5′-CTCCCTGGTACCCAGTCTCA-3′
*Shp*	F: 5′-TCCTCATGGCCTCTACCCTC-3′
	R: 5′-TCTCCCATGATAGGGCGGAA-3′
*Ibabp*	F: 5′-CAGGAGACGTGATTGAAAGGG-3′
	R: 5′-GCCCCCAGAGTAAGACTGGG-3′
*Cyp7a1*	F: 5′-TGGGGCCTGAGTTTCATCAC-3′
	R: 5′-CGAGAGCATGTCGAAACTTCC-3′
*Pparγ1*	F: 5′-GTGAGACCAACAGCCTGACG-3′
	R: 5′-ACAGACTCGGCACTCAATGG-3′
*Gapdh*	F: 5′-CACTTGAAGGGTGGAGCCAA-3′
	R: 5′-AGTGATGGCATGGACTGTGG-3′

F = forward; R = reverse. *Fxrα1/2* = farnesoid X receptor; *Shp* = small heterodimer protein; *Ibabp* = ileal bile acid-binding protein; *Cyp7a1* = cholesterol 7 alpha-hydroxylase; *Pparγ* 1 = peroxisome proliferator-activated receptor-γ1; *Gapdh* = glyceraldehyde dehydrogenase phosphate.

## Abbreviations

APC	Adenomatous polyposis Coli
αMCA	α-muricholic acid
βMCA	β-muricholic acid
BA	Bile acid
CA	Cholic acid
CDCA	Chenodeoxycholic acid
CNTL	Control diet
CYP7A1	Cytochrome P450 7A1
DCA	Deoxycholic acid
FGF15/19	Fibroblast growth factor 15/19
FGFR4	Fibroblast growth factor receptor-4
FXR	Farnesoid X receptor
FXRE	Farnesoid X receptor element
G	Glycine conjugated
HNF4α	Hepatocyte nuclear factor 4α
LA	Linoleic acid
IBABP	Ileac bile acid-binding protein
LCA	Lithocholic acid
MDCA	Murideoxycholic acid
MYTG	Methionine-tyrosine-threonine-glycine
LRH-1	Liver-related homolog-1
*n*-6HFD	Omega-6 high-fat diet
NAFLD	Nonalcoholic fatty liver disease
PPARγ1−2	Peroxisome proliferator-activated receptor-γ1 and -γ2
PUFA	Polyunsaturated fatty acid
SFA	Saturated fatty acid
SHP	Small heterodimer protein
T	Taurine conjugated
T-CA	Taurine-cholic acid
T-CDCA	Taurine-chenodeoxycholic acid
T-βMCA	Taurine-β-muricholic acid
UDCA	Ursodeoxycholic acid
WT	Wild type
%E	Percent energy

## References

[1] Blasbalg T.L., Hibbeln J.R., Ramsden C.E., Majchrzak S.F., Rawlings R.R. (2011). Changes in consumption of omega-3 and omega-6 fatty acids in the United States during the 20th century. Am. J. Clin. Nutr..

[2] Kritchevsky D. (1998). History of recommendations to the public about dietary fat. J. Nutr..

[3] Eckel R.H., Jakicic J.M., Jamy D., De Jesus J.M., Miller N.H., Hubbard V.S., Lee I.-M., Lichtenstein A.H., Loria C.M., Millen B.E. (2014). 2013 AHA/ACC Guideline on Lifestyle Management to Reduce Cardiovascular Risk: A Report of the American College of Cardiology/American Heart Association Task Force on Practice Guidelines. J. Am. Coll. Cardiol..

[4] Tjonneland A., Overvad K., Bergmann M.M., Nagel G., Linseisen J., Hallmans G., Palmqvist R., Sjodin H., Hagglund G., Berglund G. (2009). Linoleic acid, a dietary n-6 polyunsaturated fatty acid, and the aetiology of ulcerative colitis: A nested case-control study within a European prospective cohort study. Gut.

[5] Ueda Y., Kawakami Y., Kunii D., Okada H., Azuma M., Le D.S.N., Yamamoto S. (2008). Elevated concentrations of linoleic acid in erythrocyte membrane phospholipids in patients with inflammatory bowel disease. Nutr. Res..

[6] Pickens C.A., Pereira M.D.F.A., Fenton J.I. (2017). Long-chain ω-6 plasma phospholipid polyunsaturated fatty acids and association with colon adenomas in adult men. Eur. J. Cancer Prev..

[7] Torres-Castillo N., Silva-Gómez J.A., Campos-Perez W., Barron-Cabrera E., Hernandez-Cañaveral I., Garcia-Cazarin M., Marquez-Sandoval Y., Gonzalez-Becerra K., Barron-Gallardo C., Martinez-Lopez E. (2018). High Dietary ω-6:ω-3 PUFA Ratio Is Positively Associated with Excessive Adiposity and Waist Circumference. Obes. Facts.

[8] Dong Y., Zhou J., Zhu Y., Luo L., He T., Hu H., Liu H., Zhang Y., Luo D., Xu S. (2017). Abdominal obesity and colorectal cancer risk: Systematic review and meta-analysis of prospective studies. Biosci. Rep..

[9] Araya J., Rodrigo R., Videla L.A., Thielemann L., Orellana M., Pettinelli P., Poniachik J. (2004). Increase in long-chain polyunsaturated fatty acid n−6/n−3 ratio in relation to hepatic steatosis in patients with non-alcoholic fatty liver disease. Clin. Sci..

[10] Dermadi D., Valo S., Ollila S., Soliymani R., Sipari N., Pussila M., Sarantaus L., Linden J., Baumann M., Nyström M. (2017). Western Diet Deregulates Bile Acid Homeostasis, Cell Proliferation, and Tumorigenesis in Colon. Cancer Res..

[11] Girardin M., Hadengue A., Frossard J.-L. (2018). High prevalence of cholestasis, with increased conjugated bile acids in inflammatory bowel diseases patients. World J. Clin. Cases.

[12] Mouzaki M., Wang A.Y., Bandsma R., Comelli E.M., Arendt B.M., Zhang L., Fung S., Fischer S.E., McGilvray I.G., Allard J.P. (2016). Bile Acids and Dysbiosis in Non-Alcoholic Fatty Liver Disease. PLoS ONE.

[13] Pandak W.M., Kakiyama G. (2019). The acidic pathway of bile acid synthesis: Not just an alternative pathway. Liver Res..

[14] Li-Hawkins J., Gåfvels M., Olin M., Lund E.G., Andersson U., Schuster G., Björkhem I., Russell D.W., Eggertsen G. (2002). Cholic acid mediates negative feedback regulation of bile acid synthesis in mice. J. Clin. Investig..

[15] Takahashi S., Fukami T., Masuo Y., Brocker C.N., Xie C., Krausz K.W., Wolf C.R., Henderson C.J., Gonzalez F.J. (2016). Cyp2c70 is responsible for the species difference in bile acid metabolism between mice and humans. J. Lipid Res..

[16] Rudling M. (2016). Understanding mouse bile acid formation: Is it time to unwind why mice and rats make unique bile acids?. J. Lipid Res..

[17] Staley C., Weingarden A.R., Khoruts A., Sadowsky M.J. (2016). Interaction of gut microbiota with bile acid metabolism and its influence on disease states. Appl. Microbiol. Biotechnol..

[18] Wahlström A., Sayin S.I., Marschall H.-U., Bäckhed F. (2016). Intestinal Crosstalk between Bile Acids and Microbiota and Its Impact on Host Metabolism. Cell Metab..

[19] Sayin S.I., Wahlström A., Felin J., Jäntti S., Marschall H.U., Bamberg K., Angelin B., Hyötyläinen T., Orešič M., Bäckhed F. (2013). Gut Microbiota Regulates Bile Acid Metabolism by Reducing the Levels of Tauro-beta-muricholic Acid, a Naturally Occurring FXR Antagonist. Cell Metab..

[20] Li F., Jiang C., Krausz K.W., Li Y., Albert I., Hao H., Fabre K.M., Mitchell J.B., Patterson A.D., Gonzalez F.J. (2013). Microbiome remodelling leads to inhibition of intestinal farnesoid X receptor signalling and decreased obesity. Nat. Commun..

[21] Kitahara M., Takamine F., Imamura T., Benno Y. (2000). Assignment of Eubacterium sp. VPI 12708 and related strains with high bile acid 7alpha-dehydroxylating activity to Clostridium scindens and proposal of Clostridium hylemonae sp. nov., isolated from human faeces. Int. J. Syst. Evol. Microbiol..

[22] Modica S., Gadaleta R.M., Moschetta A. (2010). Deciphering the nuclear bile acid receptor FXR paradigm. Nucl. Recept. Signal..

[23] Kliewer S.A., Mangelsdorf D.J. (2015). Bile Acids as Hormones: The FXR-FGF15/19 Pathway. Dig. Dis..

[24] Inagaki T., Choi M., Moschetta A., Peng L., Cummins C.L., McDonald J.G., Luo G., Jones S.A., Goodwin B., Richardson J.A. (2005). Fibroblast growth factor 15 functions as an enterohepatic signal to regulate bile acid homeostasis. Cell Metab..

[25] Makishima M., Okamoto A.Y., Repa J.J., Tu H., Learned R.M., Luk A., Hull M.V., Lustig K.D., Mangelsdorf D.J., Shan B. (1999). Identification of a Nuclear Receptor for Bile Acids. Science.

[26] Parks D.J., Blanchard S.G., Bledsoe R.K., Chandra G., Consler T.G., Kliewer S.A., Stimmel J.B., Willson T.M., Zavacki A.M., Moore D.D. (1999). Bile Acids: Natural Ligands for an Orphan Nuclear Receptor. Science.

[27] Wang H., Chen J., Hollister K., Sowers L.C., Forman B.M. (1999). Endogenous Bile Acids Are Ligands for the Nuclear Receptor FXR/BAR. Mol. Cell.

[28] Mueller M., Thorell A., Claudel T., Jha P., Koefeler H., Lackner C., Hoesel B., Fauler G., Stojakovic T., Einarsson C. (2015). Ursodeoxycholic acid exerts farnesoid X receptor-antagonistic effects on bile acid and lipid metabolism in morbid obesity. J. Hepatol..

[29] Fang S., Suh J.M., Reilly S.M., Yu E., Osborn O., Lackey D., Yoshihara E., Perino A., Jacinto S., Lukasheva Y. (2015). Intestinal FXR agonism promotes adipose tissue browning and reduces obesity and insulin resistance. Nat. Med..

[30] Fu T., Coulter S., Yoshihara E., Oh T.G., Fang S., Cayabyab F., Zhu Q., Zhang T., Leblanc M., Liu S. (2019). FXR Regulates Intestinal Cancer Stem Cell Proliferation. Cell.

[31] Zhang Y., Kast-Woelbern H.R., Edwards P.A. (2002). Natural Structural Variants of the Nuclear Receptor Farnesoid X Receptor Affect Transcriptional Activation. J. Biol. Chem..

[32] Renga B., Mencarelli A., Migliorati M., Cipriani S., D’Amore C., Distrutti E., Fiorucci S. (2011). SHP-dependent and -independent induction of peroxisome proliferator-activated receptor-γ by the bile acid sensor farnesoid X receptor counter-regulates the pro-inflammatory phenotype of liver myofibroblasts. Inflamm. Res..

[33] Romagnolo D.F., Donovan M.G., Doetschman T.C., Selmin O.I. (2019). n-6 Linoleic Acid Induces Epigenetics Alterations Associated with Colonic Inflammation and Cancer. Nutrients.

[34] De Wit N., Derrien M., Bosch-Vermeulen H., Oosterink E., Keshtkar S., Duval C., Bosch J.D.V.-V.D., Kleerebezem M., Müller M., Van Der Meer R. (2012). Saturated fat stimulates obesity and hepatic steatosis and affects gut microbiota composition by an enhanced overflow of dietary fat to the distal intestine. Am. J. Physiol. Liver Physiol..

[35] Li J., Dawson P.A. (2019). Animal models to study bile acid metabolism. Biochim. et Biophys. Acta Mol. Basis Dis..

[36] Kir S., Zhang Y., Gerard R.D., Kliewer S.A., Mangelsdorf D.J. (2012). Nuclear Receptors HNF4α and LRH-1 Cooperate in RegulatingCyp7a1 in Vivo. J. Biol. Chem..

[37] Innes J.K., Calder P.C. (2018). Omega-6 fatty acids and inflammation. Prostaglandins Leukot. Essent. Fat. Acids.

[38] Patterson E., Wall R., Fitzgerald G.F., Ross R.P., Stanton C. (2012). Health Implications of High Dietary Omega-6 Polyunsaturated Fatty Acids. J. Nutr. Metab..

[39] Singh J., Hamid R., Reddy B.S. (1997). Dietary fat and colon cancer: Modulating effect of types and amount of dietary fat on ras-p21 function during promotion and progression stages of colon cancer. Cancer Res..

[40] Singh J., Hamid R., Reddy B.S. (1997). Dietary fat and colon cancer: Modulation of cyclooxygenase-2 by types and amount of dietary fat during the postinitiation stage of colon carcinogenesis. Cancer Res..

[41] Rao C.V., Hirose Y., Indranie C., Reddy B.S. (2001). Modulation of experimental colon tumorigenesis by types and amounts of dietary fatty acids. Cancer Res..

[42] Tang F.-Y., Pai M.-H., Chiang E.-P.I. (2012). Consumption of high-fat diet induces tumor progression and epithelial–mesenchymal transition of colorectal cancer in a mouse xenograft model. J. Nutr. Biochem..

[43] Patrone V., Minuti A., Lizier M., Miragoli F., Lucchini F., Trevisi E., Rossi F., Callegari M.L. (2018). Differential effects of coconut versus soy oil on gut microbiota composition and predicted metabolic function in adult mice. BMC Genom..

[44] Yue F., Cheng Y., Breschi A., Vierstra J., Wu W., Ryba T., Sandstrom R., Ma Z., Davis C., The Mouse ENCODE Consortium (2014). A comparative encyclopedia of DNA elements in the mouse genome. Nat. Cell Biol..

[45] Ghoneim R.H., Sock E.T.N., Lavoie J.-M., Piquette-Miller M. (2015). Effect of a high-fat diet on the hepatic expression of nuclear receptors and their target genes: Relevance to drug disposition. Br. J. Nutr..

[46] Cariello M., Piglionica M., Gadaleta R.M., Moschetta A. (2019). The Enterokine Fibroblast Growth Factor 15/19 in Bile Acid Metabolism. Handbook of Experimental Pharmacology.

[47] Jeyapal S., Kona S.R., Mullapudi S.V., Putcha U.K., Gurumurthy P., Ibrahim A. (2018). Substitution of linoleic acid with α-linolenic acid or long chain n-3 polyunsaturated fatty acid prevents Western diet induced nonalcoholic steatohepatitis. Sci. Rep..

[48] Xie G., Wang X., Huang F., Zhao A., Chen W., Yan J., Zhang Y., Lei S., Ge K., Zheng X. (2016). Dysregulated hepatic bile acids collaboratively promote liver carcinogenesis. Int. J. Cancer.

[49] Lee F.Y., Lee H., Hubbert M.L., Edwards P.A., Zhang Y. (2006). FXR, a multipurpose nuclear receptor. Trends Biochem. Sci..

[50] Song X., Chen Y., Valanejad L., Kaimal R., Yan B., Stoner M., Deng R. (2013). Mechanistic insights into isoform-dependent and species-specific regulation of bile salt export pump by farnesoid X receptor. J. Lipid Res..

[51] Grober J., Zaghini I., Fujii H., Jones S.A., Kliewer S.A., Willson T.M., Ono T., Besnard P. (1999). Identification of a Bile Acid-responsive Element in the Human Ileal Bile Acid-binding Protein Gene. J. Biol. Chem..

[52] Neimark E., Chen F., Li X., Shneider B.L. (2004). Bile acid-induced negative feedback regulation of the human ileal bile acid transporter. Hepatology.

[53] Chow E.C., Magomedova L., Quach H.P., Patel R., Durk M.R., Fan J., Maeng H.-J., Irondi K., Anakk S., Moore D.D. (2014). Vitamin D Receptor Activation Down-regulates the Small Heterodimer Partner and Increases CYP7A1 to Lower Cholesterol. Gastroenterology.

[54] Ishizawa M., Akagi D., Makishima M. (2018). Lithocholic Acid Is a Vitamin D Receptor Ligand That Acts Preferentially in the Ileum. Int. J. Mol. Sci..

[55] Degirolamo C., Rainaldi S., Bovenga F., Murzilli S., Moschetta A. (2014). Microbiota Modification with Probiotics Induces Hepatic Bile Acid Synthesis via Downregulation of the Fxr-Fgf15 Axis in Mice. Cell Rep..

[56] Zhang X., Osaka T., Tsuneda S. (2015). Bacterial metabolites directly modulate farnesoid X receptor activity. Nutr. Metab..

[57] Duan Y., Zhang F., Yuan W., Wei Y., Wei M., Zhou Y., Yang Y., Chang Y., Wu X. (2019). Hepatic cholesterol accumulation ascribed to the activation of ileum Fxr-Fgf15 pathway inhibiting hepatic Cyp7a1 in high-fat diet-induced obesity rats. Life Sci..

[58] Kübeck R., Bonet-Ripoll C., Hoffmann C., Walker A., Müller V.M., Schüppel V.L., Lagkouvardos I., Scholz B., Engel K.-H., Daniel H. (2016). Dietary fat and gut microbiota interactions determine diet-induced obesity in mice. Mol. Metab..

[59] Goodwin B., Jones S.A., Price R.R., Watson M.A., McKee D.D., Moore L.B., Galardi C., Wilson J.G., Lewis M.C., Roth M.E. (2000). A Regulatory Cascade of the Nuclear Receptors FXR, SHP-1, and LRH-1 Represses Bile Acid Biosynthesis. Mol. Cell.

[60] Fiorucci S., Rizzo G., Antonelli E., Renga B., Mencarelli A., Riccardi L., Morelli A., Pruzanski M., Pellicciari R. (2005). Cross-Talk between Farnesoid-X-Receptor (FXR) and Peroxisome Proliferator-Activated Receptor γ Contributes to the Antifibrotic Activity of FXR Ligands in Rodent Models of Liver Cirrhosis. J. Pharmacol. Exp. Ther..

[61] Fajas L., Auboeuf D., Raspé E., Schoonjans K., Lefebvre A.-M., Saladin R., Najib J., Laville M., Fruchart J.-C., Deeb S. (1997). The Organization, Promoter Analysis, and Expression of the Human PPARγ Gene. J. Biol. Chem..

[62] Correia J.C., Massart J., De Boer J.F., Porsmyr-Palmertz M., Martinez-Redondo V., Agudelo L.Z., Sinha I., Meierhofer D., Ribeiro V., Björnholm M. (2015). Bioenergetic cues shift FXR splicing towards FXRα2 to modulate hepatic lipolysis and fatty acid metabolism. Mol. Metab..

[63] Alvarez-Sola G., Uriarte I., Latasa M.U., Fernandez-Barrena M.G., Urtasun R., Elizalde M., Barcena-Varela M., Jiménez M., Chang H.C., Barbero R. (2017). Fibroblast growth factor 15/19 (FGF15/19) protects from diet-induced hepatic steatosis: Development of an FGF19-based chimeric molecule to promote fatty liver regeneration. Gut.

[64] Lv H., Ren D., Yan W., Wang Y., Liu H., Shen M. (2020). Linoleic acid inhibits Lactobacillus activity by destroying cell membrane and affecting normal metabolism. J. Sci. Food Agric..

[65] Jenkins J.K., Courtney P.D. (2003). Lactobacillusgrowth and membrane composition in the presence of linoleic or conjugated linoleic acid. Can. J. Microbiol..

[66] De Weirdt R., Coenen E., Vlaeminck B., Fievez V., Abbeele P.V.D., Van De Wiele T. (2013). A simulated mucus layer protects Lactobacillus reuteri from the inhibitory effects of linoleic acid. Benef. Microbes.

[67] Devkota S., Wang Y., Musch M.W., Leone V., Fehlner-Peach H., Nadimpalli A., Antonopoulos D.A., Jabri B., Chang E.B. (2012). Dietary-fat-induced taurocholic acid promotes pathobiont expansion and colitis in Il10−/− mice. Nat. Cell Biol..

[68] Suzuki R., Miyamoto S., Yasui Y., Sugie S., Tanaka T. (2007). Global gene expression analysis of the mouse colonic mucosa treated with azoxymethane and dextran sodium sulfate. BMC Cancer.

[69] Larionov A.A., Krause A., Miller W.R. (2005). A standard curve based method for relative real time PCR data processing. BMC Bioinform..

[70] Batta A.K., Salen G., Rapole K.R., Batta M., Batta P., Alberts D., Earnest D. (1999). Highly simplified method for gas-liquid chromatographic quantitation of bile acids and sterols in human stool. J. Lipid Res..

[71] Want E.J., Coen M., Masson P., Keun H.C., Pearce J.T.M., Reily M.D., Robertson D.G., Rohde C.M., Holmes E., Lindon J.C. (2010). Ultra Performance Liquid Chromatography-Mass Spectrometry Profiling of Bile Acid Metabolites in Biofluids: Application to Experimental Toxicology Studies. Anal. Chem..

[72] Selmin O.I., Fang C., Lyon A.M., Doetschman T.C., Thompson P.A., Martinez J.D., Smith J.W., Lance P.M., Romagnolo D.F. (2015). Inactivation of Adenomatous Polyposis Coli Reduces Bile Acid/Farnesoid X Receptor Expression through Fxr gene CpG Methylation in Mouse Colon Tumors and Human Colon Cancer Cells. J. Nutr..

[73] Sheng L., Jena P.K., Liu H.-X., Kalanetra K.M., Gonzalez F.J., French S.W., Krishnan V.V., Mills D.A., Wan Y.-J.Y. (2017). Gender Differences in Bile Acids and Microbiota in Relationship with Gender Dissimilarity in Steatosis Induced by Diet and FXR Inactivation. Sci. Rep..

[74] Shin D.-J., Osborne T.F. (2009). FGF15/FGFR4 Integrates Growth Factor Signaling with Hepatic Bile Acid Metabolism and Insulin Action. J. Biol. Chem..

[75] Fiorucci S., Biagioli M., Zampella A., Distrutti E. (2018). Bile Acids Activated Receptors Regulate Innate Immunity. Front. Immunol..

